# IVIG Associated Aseptic Meningitis in a Renal Transplant Patient

**DOI:** 10.1155/2017/6962150

**Published:** 2017-05-23

**Authors:** Tamara Wanigasekera, Rachel J. Grainger, Donal J. Sexton, Colm Magee

**Affiliations:** ^1^Transplant, Urology and Nephrology Directorate, Beaumont Hospital, Dublin 9, Ireland; ^2^Department of Clinical Microbiology, Beaumont Hospital, Dublin 9, Ireland

## Abstract

The management of antibody-mediated rejection in renal transplant recipients involves plasmapheresis with IVIG. Aseptic meningitis is a rare adverse effect of IVIG therapy and is a diagnosis of exclusion. We report a case of a renal transplant patient who developed IVIG associated aseptic meningitis in the context of management of antibody-mediated rejection, four years after transplantation.

## 1. Introduction

IVIG associated meningitis is reported to occur in less than 1% of patients exposed to IVIG [1]. We have identified one prior report of IVIG associated meningitis in a renal transplant patient undergoing treatment of antibody-mediated rejection [2]. It is more frequently reported in the nontransplant setting, for example, the treatment of neuromuscular disorders and of immune deficiency. The initial presentation of IVIG associated meningitis is indistinguishable from infective meningitis.

## 2. Case Presentation

A 31-year-old male with ESKD secondary to IgA nephropathy received a deceased donor kidney transplant four years prior to presentation. Antibody-mediated rejection (ABMR) was diagnosed on the basis of typical changes on light microscopy (including peritubular capillaritis) and positive staining for C4d staining and the presence in the serum of new donor specific antibodies. The patient was on maintenance immunosuppression consisting of tacrolimus 1.5 mg twice daily, mycophenolate mofetil 750 mg twice daily, and prednisolone 5 mg once daily. He denied noncompliance and had acceptable immunosuppressant drug trough levels.

The patient received 250 mg methylprednisolone daily for three days and underwent five sessions of plasma exchange followed by IVIG (1 g/kg) at a slow rate of 54 ml/hour increasing to 108 ml/hour as per protocol with concomitant IV normal saline and paracetamol, prophylactic cotrimoxazole, and valacyclovir. The total dose received was 90 g. Less than 24 hours after the final dose of IVIG, he presented to emergency services with a severe headache, photophobia, and nuchal rigidity. He had no other neurological symptoms or signs.

Computerized tomography (CT) neuroimaging was noncontributory. Lumbar punctures on days 1 and 2 of admission yielded clear cerebrospinal fluid (CSF) with a polymorphic leukocytosis, elevated protein, and low glucose (Table 1). Gram stain and culture were negative. Serum C-reactive protein and white cell count were normal.

Broad-spectrum empiric antimicrobial therapy was started at presentation. This consisted of high dose intravenous acyclovir, vancomycin, cefotaxime, and amoxicillin.

Microbiological analysis of CSF samples was negative for culture and PCR of typical and atypical bacterial and viral causes of meningitis, as described below.

CSF samples were negative for the following:* Neisseria meningitidis*,* Streptococcus pneumoniae*,* Cryptococcus neoformans*,* Listeria monocytogenes*, cytomegalovirus (CMV), enterovirus, Herpes simplex virus, and Varicella zoster virus as well as acid fast bacilli (Table 2). Serum testing for HIV, hepatitis B, and hepatitis C was also negative (Table 3).

As microbiological tests were negative, initial empirical antimicrobials were discontinued by day seven of admission and the patient was managed supportively with intravenous fluids, analgesia, and antiemetics. IVIG treatment had been completed prior to this presentation and the patient did not require further doses. By day five, the patient was asymptomatic. Four weeks after discharge the patient remained well and was continued on a higher dose of maintenance immunosuppression during this time, with stable graft function.

## 3. Discussion

As with reported cases of IVIG associated meningitis, this patient's presentation mimicked infective meningitis. Like other reported cases [1–8], he had signs of meningism on examination and had a polymorphic leukocytosis with raised protein on CSF analysis. He also presented within 48 hours of receiving IVIG at a higher doses (1 g/kg) than that used in IVIG replacement in hypogammaglobulinaemia.

When the above case is compared to the prior case of IVIG associated meningitis in a renal transplant patient undergoing treatment of antibody-mediated rejection (Wright et al. [2]) the biochemical picture is similar to differences in clinical presentation.

In the case described by Wright et al. [2] the patient presented with a mild headache, nuchal rigidity, and pyrexia 48 hours after IVIG administration, where the total dose used was higher (120 g). Our patient presented within 24 hours with a severe headache, photophobia, and nuchal rigidity and was afebrile. The main clinical difference was that while our patient had no focal neurological signs, the patient in the case described by Wright et al. developed an abducens nerve palsy on day eight of her admission [2] and took a week later to recover in terms of symptoms. This resolved two weeks later and was postulated to be due to perineuritis [2]. In terms of investigations, both patients had noncontributory imaging and a polymorphonuclear leukocytosis on CSF sampling. In both cases, white cell counts of over 2000 cells/mL, consisting of greater than 70% polymorphs, were found on microscopy. Overall, although both cases were similar in terms of investigation results, our patient received a lower total dose with rapid onset of severe symptoms and swift resolution five days after presentation. This emphasizes the idiosyncratic nature of the IVIG induced drug reaction.

IVIG therapy is used outside the field of transplant medicine in the management of demyelinating disease, autoimmune diseases, and primary and secondary immune deficiency. It is a blood derived product composed of polyclonal human immunoglobulin from up to 15,000 donors. The exact handling of IVIG at the blood brain barrier is incompletely characterised. Murine modeling demonstrates that less than 0.01% crosses the blood brain barrier [9]. The exact mechanism of IVIG associated meningitis is poorly understood and it is thought to be more complex than direct meningeal irritation. Postulated mechanisms include cerebral vasospasm [10], neutrophil activation [10, 11], and hypersensitivity reaction [10]. Neutrophil activation by IVIG has been demonstrated in vivo by antibodies found in IVIG [10] and directly via the Fc*γ*RIII neutrophil surface membrane receptor [11]. Cerebral artery vasospasm following IVIG therapy has been demonstrated using transcranial Doppler in small numbers of patients presenting with encephalopathy following IVIG therapy in Guillain-Barre syndrome [12] but may also be a feature of the underlying neurological condition.

There are no established risk factors for anticipating development of IVIG associated aseptic meningitis, although some case reports have observed a background history of migraine [6]. It is common practice to use a slow rate with concurrent IV hydration and antihistamine before medication to minimise the occurrence of adverse effects in IVIG administration.

IVIG is sometimes administered to renal transplant patients in the setting of antibody-mediated rejection. IVIG associated meningitis may initially present in a similar manner to the infusion reaction headache that is commonly observed. It should be a rare diagnosis of exclusion and there are very few reported cases on the renal transplant population. It is important to ensure that typical and atypical organisms are considered when investigating meningitis, especially in the immune-compromised patient. It is a significant complication to be aware of, due to its morbidity and because its presentation mimics the signs and CSF findings of infective meningitis.

## Figures and Tables

**Table 1:** CSF composition.

	Day 1	Day 2
White cell count/mL	3846	1037
Differential	90% polymorphs	79% polymorphs, 16% lymphocytes
Red cell count/mL	50	6
Protein (mg/dL)	139.5	40.0
Glucose (mmol/L)	2.1	3.0

mL = milliliters, mg/dL = milligrams per decilitre, and mmol/L = millimoles per deciliter.

**Table 2:** Tests of CSF.

Investigation	Result
Gram stain and culture	Negative
Pneumococcal PCR	Negative
Meningococcal PCR	Negative
Listeria monocytogenes PCR	Negative
Acid fast bacilli	Negative
TB culture	Negative
Herpes simplex virus 1&2 PCR	Negative
Varicella zoster virus PCR	Negative
Enterovirus PCR	Negative
Cytomegalovirus PCR	Negative
Cryptococcal antigen	Negative

**Table 3:** Tests of peripheral blood.

Investigation	Result
Gram stain and culture	Negative

Cryptococcal antigen	Negative

Meningococcal PCR	Negative

Pneumococcal PCR	Negative

Hepatitis C PCR	Negative

Hepatitis B surface antigen	Negative
Hepatitis B antibodies	
Hepatitis C antigen	
HIV1 + 2	
